# Body Mass Index Does Not Predict High Miller–Payne Response to Neoadjuvant Chemotherapy in Stage II–III Breast Cancer: A Single-Center Retrospective Cohort Study of 647 Patients

**DOI:** 10.3390/jcm15093423

**Published:** 2026-04-30

**Authors:** Hasan Acik, Serdar Arici

**Affiliations:** 1 Department of Internal Medicine, Faculty of Medicine, Okan University, 34947 Istanbul, Türkiye; 2 Department of Medical Oncology, Faculty of Medicine, Okan University, 34947 Istanbul, Türkiye

**Keywords:** neoadjuvant chemotherapy, Miller–Payne response, body mass index, HER2, Ki-67

## Abstract

**Background**: Neoadjuvant chemotherapy (NACT) response in breast cancer is primarily influenced by tumor biology; however, the independent role of host-related factors such as body mass index (BMI) remains unclear. This study evaluated clinicopathological predictors of a high Miller–Payne response after neoadjuvant chemotherapy in patients with stage II–III non-metastatic breast cancer, with particular focus on the role of body mass index. **Methods**: In this retrospective cohort study, 647 patients with stage II–III non-metastatic breast cancer treated with standard NACT between January 2010 and December 2020 at a single tertiary center were included. High pathological response was defined as Miller–Payne Grades 4–5. Clinical and tumor-related variables were analyzed, including age, menopausal status, body mass index, clinical stage, tumor grade, estrogen receptor status, progesterone receptor status, human epidermal growth factor receptor 2 status, and Ki-67 proliferation index. Univariate and multivariable logistic regression analyses were performed to determine independent predictors of high pathological response. **Results**: A total of 647 patients were included (mean age: 49.18 ± 10.94 years; mean BMI: 28.45 ± 5.45 kg/m^2^), of whom 47.45% were postmenopausal. High Miller–Payne response (Grades 4–5) was observed in 40.96% of patients. High response was significantly associated with clinical stage (*p* = 0.034), tumor grade (*p* < 0.001), HER2 status (*p* < 0.001), ER status (*p* < 0.001), PR status (*p* < 0.001), and Ki-67 levels (*p* < 0.001). Median Ki-67 was higher in the high-response group (35.00% [IQR: 20.00–62.75] vs. 25.00% [IQR: 15.00–45.75], *p* < 0.001). No significant associations were observed for age (*p* = 0.299), BMI (*p* = 0.874), or menopausal status (*p* = 0.289). In multivariable analysis, HER2 positivity (adjusted OR = 3.71, 95% CI: 2.23–6.20, *p* < 0.001) and Ki-67 (adjusted OR = 1.01 per 1% increase, 95% CI: 1.00–1.02, *p* = 0.009) were independently associated with high response, whereas ER positivity (adjusted OR = 0.50, 95% CI: 0.25–0.98, *p* = 0.043) and postmenopausal status (adjusted OR = 0.37, 95% CI: 0.17–0.81, *p* = 0.013) were inversely associated. BMI was not independently associated with response (*p* = 0.964). **Conclusions**: Response to neoadjuvant chemotherapy appears to be mainly determined by tumor biology, particularly receptor status and proliferative activity, rather than anthropometric measures such as body mass index.

## 1. Introduction

Neoadjuvant chemotherapy (NACT) is integral to the treatment of stage II–III breast cancer, allowing tumor downstaging, facilitating breast-conserving surgery, and assessing tumor chemosensitivity. The pathological response after NACT correlates with long-term outcomes, particularly in HER2-positive and triple-negative breast cancers. Pathologic complete response (pCR) serves as a surrogate endpoint; however, semi-quantitative regression systems, such as Miller–Payne grading, provide a granular assessment of tumor cell reduction. The Miller–Payne system evaluates the decrease in tumor cellularity between pre-treatment biopsy and post-treatment surgical specimens, stratifying the response from no change (Grade 1) to complete disappearance of invasive tumor cells (Grade 5) [1]. Tumor biology remains a key determinant of the pathological response to NACT. High proliferative activity, assessed using Ki-67, correlates with improved chemotherapy responsiveness in all cohorts [2]. Similarly, HER2 overexpression links to higher pCR rates with anti-HER2-directed regimens [3]. Conversely, hormone receptor–positive luminal tumors show lower pathological regression rates, reflecting intrinsic differences in chemosensitivity [2]. Host-related factors, particularly obesity, have been investigated as potential modifiers of chemotherapy response. Obesity may contribute to tumor behavior through increased aromatase activity, higher circulating estrogen levels in postmenopausal women, chronic inflammation, altered adipokine signaling, and insulin resistance. These metabolic alterations may influence tumor proliferation and therapeutic sensitivity of the tumor. Studies exploring the relationship between body mass index (BMI) and NACT response have shown inconsistent findings. Some studies suggest that a higher BMI is associated with lower pCR rates [3,4], while others report no independent association after adjusting for tumor subtype and treatment variables [5,6,7]. Few large-scale studies have examined anthropometric variables using the Miller–Payne regression system. Furthermore, comparative evaluations of anthropometric, clinical, and tumor biological factors within a single large cohort remain limited. Clarifying whether host-related variables independently predict a high Miller–Payne response beyond established biological markers is essential for refining prognostic stratification and avoiding the overinterpretation of anthropometric metrics.

Therefore, the present study aimed to evaluate the clinicopathologic predictors of a high Miller–Payne response (Grades 4–5) in a retrospective cohort of 647 patients with stage II–III breast cancer treated with NACT. Given the biologic links between adiposity, aromatase activity, insulin resistance, chronic inflammation, and altered adipokine signaling, BMI was examined in the present study as the principal host-related exposure of interest. We specifically aimed to determine whether baseline BMI independently predicted high Miller–Payne response beyond established tumor-intrinsic determinants such as hormone receptor status, HER2 status, and Ki-67.

## 2. Materials and Methods

### 2.1. Study Design and Setting

This retrospective cohort study was conducted at the Okmeydani Training and Research Hospital, a tertiary academic referral center.

### 2.2. Study Population

All consecutive patients diagnosed with clinically stage II–III invasive breast carcinoma who were evaluated for neoadjuvant chemotherapy (NACT) at Okmeydani Training and Research Hospital between January 2010 and December 2020 were retrospectively screened for eligibility.

Inclusion criteria were defined as follows: (i) histologically confirmed invasive breast carcinoma based on core needle biopsy; (ii) clinical stage II or III disease at initial diagnosis according to the AJCC staging system; (iii) receipt of standard neoadjuvant chemotherapy according to institutional protocols; (iv) completion of the planned NACT regimen without premature discontinuation due to non-oncologic reasons; and (v) availability of complete pathological response assessment using the Miller–Payne grading system based on paired pre-treatment biopsy and post-treatment surgical specimens.

Exclusion criteria included: (i) presence of distant metastatic disease (stage IV, M1) at initial diagnosis; (ii) prior systemic therapy, radiotherapy, or surgical treatment for breast cancer before initiation of NACT; (iii) lack of definitive surgical resection following NACT; (iv) missing or incomplete key clinicopathologic data, including receptor status (ER, PR, HER2), Ki-67 index, or BMI; (v) absence of documented Miller–Payne grading in the pathology report; and (vi) patients with non-invasive histologies or rare subtypes not amenable to standardized response assessment.

During the study period, a total of approximately 810 patients were initially identified. After exclusion of patients with metastatic disease at diagnosis (*n* = 78), incomplete treatment or missing essential clinical data (*n* = 52), and absence of evaluable pathological response data (*n* = 33), a final cohort of 647 patients meeting all eligibility criteria was included in the analysis.

### 2.3. Clinical and Pathologic Variables

Baseline demographic and clinical data included age at diagnosis, menopausal status, body mass index (BMI, kg/m^2^), clinical tumor stage (cT and cN), comorbid conditions, and family histories. BMI was calculated using the measured height and weight prior to the initiation of neoadjuvant therapy. Baseline weight and height recorded before initiation of neoadjuvant therapy were used to calculate BMI as weight in kilograms divided by height in meters squared (kg/m^2^). BMI was evaluated as a surrogate measure of host adiposity and entered into the regression analyses as a continuous variable; additional interaction analyses were performed to explore whether the association between BMI and treatment response differed according to menopausal status and receptor-defined subgroups. Tumor biological characteristics were extracted from formal pathology reports and included estrogen receptor (ER) and progesterone receptor (PR) expression status, HER2 status determined by immunohistochemistry with fluorescence in situ hybridization (FISH) confirmation when indicated according to institutional standard protocols, as well as Ki-67 proliferation index, and histological tumor grade.

### 2.4. Neoadjuvant Chemotherapy

All patients received standard NACT regimens according to institutional protocols and contemporary international guidelines, tailored to tumor subtype and clinical characteristics. Neoadjuvant chemotherapy followed institutional protocols and contemporary guideline-based practice during the study period and was predominantly based on anthracycline- and taxane-containing combinations. In routine practice, the most commonly used backbone regimens consisted of anthracycline/cyclophosphamide-based therapy (AC or EC/FEC) followed by a taxane (weekly paclitaxel or 3-weekly docetaxel), selected according to clinical stage, tumor biology, age, comorbidity profile, and treating physician judgment. In HER2-positive disease, trastuzumab-containing therapy was incorporated according to contemporaneous standards, typically together with the taxane component, and was continued after surgery to complete a total of 1 year of anti-HER2 therapy when indicated. Carboplatin-containing regimens were used in selected high-risk cases, particularly in biologically aggressive disease, but were not routinely administered across the entire cohort.

### 2.5. Pathologic Response Assessment

Pathologic response was assessed using the Miller–Payne grading system, which evaluates the proportional reduction in tumor cellularity by comparing pre-treatment core biopsy specimens with post-treatment surgical specimens. For the purposes of this study, high pathologic response was defined as Miller–Payne Grades 4–5, whereas Grades 1–3 were categorized as low to moderate response. The primary study outcome was the achievement of a high Miller–Payne response.

### 2.6. Statistical Analysis

All statistical analyses were conducted using Python (version 3.11.5; Phyton Software Foundation, Willmington, DE, USA) with the pandas (v2.1.1), NumPy (v1.26.2), SciPy (v1.11.4), statsmodels (v0.14.0), and scikit-learn (v1.3.2) libraries. Continuous variables were summarized as mean ± standard deviation or median (interquartile range) according to distribution assessed by the Shapiro–Wilk test, while categorical variables were expressed as frequencies and percentages. Between-group comparisons (high vs. low–moderate Miller–Payne response) were performed using Student’s *t*-test or Mann–Whitney U test for continuous variables and the chi-square or Fisher’s exact test for categorical variables, as appropriate. Univariable logistic regression analyses were used to evaluate associations between individual predictors and high Miller–Payne response (Grades 4–5). Variables with *p* < 0.10 in univariable analysis were entered into a multivariable logistic regression model to identify independent predictors, and adjusted odds ratios (aORs) with 95% confidence intervals (CIs) were reported. Model discrimination was assessed using the area under the receiver operating characteristic curve (AUC). All tests were two-tailed, and statistical significance was defined as *p* < 0.05.

### 2.7. Ethical Approval

The study was conducted in accordance with the ethical principles of the Declaration of Helsinki and was approved by the Institutional Review Board of Health Sciences University and Okmeydani Training and Research Hospital. Ethics Committee approval was received (IRB approval number: 50200903-903.07.02). The requirement for informed consent was waived due to the retrospective nature of the study and anonymized data analysis.

## 3. Results

A total of 647 patients with stage II–III non-metastatic breast cancer were included in the analysis. The mean age was 49.18 ± 10.94 years, and the mean BMI was 28.45 ± 5.45 kg/m^2^. Postmenopausal women constituted 47.45% of the cohort. Clinical stage II and III disease were nearly equally distributed (50.08% and 49.92%, respectively). Invasive ductal carcinoma accounted for 89.34% of cases. HER2 positivity was observed in 26.27% of tumors, while 52.55% were ER positive and 27.20% were PR positive. High Miller–Payne response (Grades 4–5) was achieved in 40.96% of patients (Table 1).

Data are presented as mean ± standard deviation for normally distributed continuous variables and as median (interquartile range) for non-normally distributed variables. Categorical variables are expressed as a number (percentage). Normality was assessed using the Shapiro–Wilk test.

High Miller–Payne response (Grades 4–5) was significantly associated with clinical stage (*p* = 0.034), tumor grade (*p* < 0.001), HER2 status (*p* < 0.001), ER status (*p* < 0.001), PR status (*p* < 0.001), and Ki-67 level (*p* < 0.001). Stage II disease was observed in 55.09% of high responders compared with 46.60% in the low–moderate response group. Grade 3 tumors were present in 36.23% of high responders versus 14.66% in the low–moderate response group. In an exploratory analysis according to surrogate molecular subtype, high Miller–Payne response differed significantly across subtypes (*p* < 0.001). High response was observed in 68.8% of HER2-positive tumors (117/170) and 54.5% of triple-negative tumors (55/101), compared with 24.6% of luminal A-like tumors (29/118) and 24.7% of luminal B-like tumors (37/150). ER positivity (37.74% vs. 62.83%) and PR positivity (15.09% vs. 35.60%) differed significantly between response groups. Median Ki-67 was 35.00% (IQR: 20.00–62.75) in the high-response group and 25.00% (IQR: 15.00–45.75) in the low–moderate response group. Age (*p* = 0.299), BMI (*p* = 0.874), and menopausal status (*p* = 0.289) were not significantly associated with response (Table 2).

Continuous variables were compared using Student’s *t*-test or Mann–Whitney U test as appropriate based on distribution. Categorical variables were analyzed using the chi-square test. All tests were two-sided, and a *p*-value <0.05 was considered statistically significant.

Surrogate molecular subtype analysis was available for 539 patients with complete HR/HER2/Ki-67 data (238 with Miller–Payne 4–5 and 301 with Miller–Payne 1–3). Luminal A-like was defined as HR-positive/HER2-negative tumors with Ki-67 <20%, and luminal B-like as HR-positive/HER2-negative tumors with Ki-67 ≥20%. HER2-positive tumors were classified irrespective of hormone receptor status.

In univariable logistic regression analysis, tumor grade, HER2 status, ER status, PR status, clinical stage, and Ki-67 were significantly associated with high Miller–Payne response. Grade 3 tumors demonstrated an odds ratio (OR) of 7.48 (95% CI: 3.59–15.58, *p* < 0.001). HER2 positivity was associated with an OR of 5.20 (95% CI: 3.54–7.64, *p* < 0.001). Ki-67 was significantly associated per 1% increase (OR = 1.02, 95% CI: 1.01–1.03, *p* < 0.001). Clinical stage III was associated with lower odds of high response (OR = 0.71, 95% CI: 0.52–0.97, *p* = 0.034). Age, BMI, menopausal status, and histology were not statistically significant (Table 3).

Univariable logistic regression analysis was performed with high Miller–Payne response (Grades 4–5) as the dependent variable. Odds ratios (ORs) with 95% confidence intervals (CIs) were calculated. Continuous variables were entered as linear terms.

In multivariable logistic regression analysis, HER2 positivity (adjusted OR = 3.71, 95% CI: 2.23–6.20, *p* < 0.001) and Ki-67 (adjusted OR = 1.01 per 1% increase, 95% CI: 1.00–1.02, *p* = 0.009) remained independently associated with high Miller–Payne response. ER positivity (score 2 vs. ER 0) was independently associated with reduced odds of high response (adjusted OR = 0.50, 95% CI: 0.25–0.98, *p* = 0.043). Postmenopausal status was also independently associated (adjusted OR = 0.37, 95% CI: 0.17–0.81, *p* = 0.013) with response. Clinical stage, BMI, age, PR status, and histology were not independently associated with response (Table 4).

Multivariable logistic regression analysis was conducted using complete-case data. Adjusted odds ratios (aORs) with 95% confidence intervals (CIs) were reported. Variables were included based on clinical relevance and univariable screening criteria (*p* < 0.10). Model discrimination was assessed using the area under the receiver operating characteristic curve (AUC).

Multicollinearity among independent variables was assessed using the variance inflation factor (VIF) analysis. The VIF values were 3.10 for age, 1.20 for body mass index, 3.02 for menopausal status, 1.75 for estrogen receptor status, 1.60 for progesterone receptor status, 1.12 for HER2 status, and 1.14 for Ki-67. The maximum observed VIF value was 3.10.

In adjusted interaction analyses evaluating potential effect modification between BMI and key clinicopathologic variables, no statistically significant multiplicative interactions were identified. Across all interaction models, the inclusion of multiplicative BMI terms did not significantly improve model fit compared with the main-effects-only models (Table 5). Stratified effect estimates of BMI within each subgroup are presented in Supplementary Table S1.

Interaction effects were evaluated using adjusted logistic regression models including multiplicative interaction terms. Likelihood ratio (LR) tests were performed by comparing reduced and full models. BMI was modeled as a continuous variable scaled per 5 kg/m^2^ increase and centered at the cohort mean. Continuous missing values were imputed using median values, and categorical missing values were modeled as a separate category.

Stratified odds ratios were derived from adjusted logistic regression models including the corresponding interaction term. BMI was modeled per a 5 kg/m^2^ increase.

## 4. Discussion

The present study evaluated the independent determinants of high Miller–Payne response (Grades 4–5) following NACT in a large, single-center cohort of 647 patients with stage II–III non-metastatic breast cancer. The principal finding is that tumor-intrinsic biological factors—particularly HER2 positivity and proliferative activity measured by Ki-67—were the dominant independent predictors of major pathological regression, whereas BMI did not independently predict high Miller–Payne response. Furthermore, BMI did not demonstrate significant effect modification across menopausal, ER, PR, or HER2-defined subgroups. These results suggest that chemotherapy sensitivity in the neoadjuvant setting is primarily governed by tumor biology rather than host anthropometric metrics.

The clinical significance of major pathological response after NACT has been firmly established. In the pooled CTNeoBC analysis including 11,955 patients, Cortazar et al. demonstrated that achievement of pCR was associated with improved event-free and overall survival, particularly in HER2-positive and triple-negative subtypes [2]. Although the present study by Ogston et al. utilized the Miller–Payne regression system rather than strict pCR criteria, Grades 4–5 reflect ≥90% reduction in tumor cellularity and have been shown to correlate with favorable long-term outcomes [8]. More recently, the NeoSTEEP consensus emphasized standardization of neoadjuvant endpoints and reaffirmed the prognostic validity of pathological response metrics [9]. Therefore, identifying robust predictors of high Miller–Payne response is clinically relevant, not only as a surrogate of survival but also as a determinant of subsequent adjuvant treatment strategies.

In our cohort, HER2 positivity conferred a nearly fourfold increased likelihood of high Miller–Payne response. This magnitude is consistent with modern neoadjuvant trials incorporating dual HER2 blockade. The NeoSphere trial demonstrated significantly increased pCR rates with trastuzumab plus pertuzumab compared with trastuzumab alone [10]. Long-term follow-up confirmed durable event-free survival benefit [11]. Contemporary meta-analyses report pooled pCR rates exceeding 55–60% in HER2-positive disease treated with optimized regimens [8].

Similarly, proliferative activity measured by Ki-67 independently predicted high pathological regression in our study. High proliferation enhances susceptibility to cytotoxic agents targeting rapidly dividing cells. A meta-analysis by Petrelli et al. confirmed that elevated baseline Ki-67 correlates with increased pCR probability across molecular subtypes [9]. Thus, our findings reinforce the central role of intrinsic tumor biology in determining chemotherapy sensitivity.

Conversely, ER positivity was independently associated with lower odds of major pathological regression. This aligns with the CTNeoBC findings that luminal tumors demonstrate lower pCR rates [10]. However, luminal tumors derive substantial benefit from endocrine therapy, highlighting that chemotherapy response and long-term prognosis are not synonymous.

The absence of an independent association between BMI and high Miller–Payne response in our cohort contrasts with some prior studies suggesting reduced pCR in overweight or obese patients. A comprehensive meta-analysis by Wang et al., including 18,702 patients, reported lower pCR rates in patients with BMI ≥ 25 kg/m^2^ (OR 0.80) and in obese patients (OR 0.68) [12]. These findings have frequently been cited to support a negative impact of excess adiposity on chemotherapy responsiveness.

However, other analyses have reported null associations after adjusting for tumor subtype and treatment variables. Erbes et al. found no independent relationship between BMI and pCR in a real-world cohort [11]. Kogawa et al. reported heterogeneous effects depending on molecular subtype, with no consistent independent signal [5]. Similarly, Raman et al. observed that BMI alone did not predict pCR when full weight-based dosing was applied; instead, the interaction between BMI and dose reductions influenced outcomes [4].

More recent national-level data from Denmark demonstrated that obesity was associated with lower pCR rates, particularly in postmenopausal women (adjusted OR = 0.62) [13]. These discrepancies likely reflect differences in endpoint definitions, dosing practices, subtype distribution, and metabolic phenotyping.

Importantly, our study extends this discussion by demonstrating not only the absence of a main BMI effect but also the absence of significant interaction between BMI and menopausal, ER, PR, or HER2 status. This suggests that BMI does not meaningfully modify chemotherapy sensitivity across biologically defined subgroups within our cohort.

Obesity is biologically complex. Adipose tissue acts as an endocrine organ, producing inflammatory cytokines, adipokines, and estrogen via aromatase activity. Obesity-associated crown-like structures (CLS-B) have been correlated with local inflammatory signaling [14]. Experimental models demonstrate that adipocytes can promote chemotherapy resistance via drug sequestration and efflux mechanisms involving major vault protein [15]. Yet BMI is an imprecise surrogate of metabolically active adiposity. It does not distinguish visceral fat from subcutaneous fat, nor fat from lean mass. CT-based body composition analyses indicate that visceral adiposity and sarcopenia may more strongly influence chemotherapy tolerance and survival than BMI itself [16]. Additionally, insulin resistance and metabolic syndrome—rather than BMI per se—have been associated with altered neoadjuvant response [17]. Thus, the null BMI finding in our cohort does not negate a biological role of adiposity; rather, it suggests that BMI alone inadequately captures the metabolic phenotype influencing chemotherapy sensitivity.

Another critical dimension is chemotherapy dosing. Historical underdosing of obese patients contributed to inferior outcomes in earlier studies. Current ASCO guidelines recommend full weight-based dosing in obese patients [18]. Pharmacokinetic studies demonstrate comparable paclitaxel exposure when dosed by actual body weight [19]. In modern practice settings, adhering to these principles, BMI may exert less influence on pathological response.

Contemporary evidence suggests that the obesity–breast cancer interface is mediated not simply by excess body mass, but by a complex adipokine network within the tumor microenvironment. Leptin, resistin, visfatin, and related adipokines have generally been linked to proliferative, inflammatory, angiogenic, and stemness-associated signaling, whereas adiponectin has more often demonstrated anti-proliferative and metabolically protective effects. However, the clinical translation of these observations remains incomplete. In a prospective cohort of 3112 breast cancer patients, post-diagnosis circulating leptin, adiponectin, and resistin were not associated with long-term outcomes overall after multivariable adjustment, although in ER/PR-negative tumors, higher adiponectin was associated with increased breast cancer-specific mortality. Chemerin represents another obesity-related mediator of interest. Recent data indicate a biologically complex and likely context-dependent role, as serum chemerin has been reported to be elevated in breast cancer and associated with histologic grade and Ki-67, while tissue-expression studies have linked chemerin to lymph node metastasis and c-ERB2 expression despite inconsistent associations with stage and hormone receptor status. Experimental studies further suggest that chemerin may also exert antitumor effects through recruitment of NK and T-cell effectors into the tumor microenvironment. Collectively, these data support the concept that functional adipokine signaling may capture the obesity-related biological milieu more accurately than BMI alone, which may partly explain why BMI was not an independent predictor in the present cohort [20].

Beyond systemic metabolic biology, the recent breast cancer literature also highlights the importance of refined locoregional staging and biologic stratification. Indocyanine green fluorescence is emerging as a valuable sentinel lymph node tracer because it enables real-time lymphatic visualization without radiation exposure. Recent reviews and pooled analyses indicate that ICG is superior to blue dye and at least comparable to radioisotope-based mapping, while the FLUORO trial reported SLN identification rates of 97.8% for ICG and 98.3% for technetium-99m, with metastatic SLN detection rates of 95.2% and 98.8%, respectively. In the post-neoadjuvant setting, early series also suggest that ICG-guided axillary staging is feasible, with an identification rate of 91.5% and no major safety signal. At the translational level, epithelial-to-mesenchymal transition is particularly relevant in metaplastic breast cancer, a rare and aggressive subtype characterized by epithelial–mesenchymal plasticity, chemoresistance, and unfavorable outcomes. Recent work suggests that TGF-β-driven EMT and stabilization of hybrid EMT states, rather than a fixed fully mesenchymal phenotype, may be central to metaplastic tumorigenesis, while CCN6 loss and Wnt/β-catenin–EZH2 signaling appear to promote EMT-associated progression. Finally, although distinct from the invasive disease analyzed in the present cohort, the clinical significance of HER2 expression in DCIS further illustrates the primacy of tumor biology. HER2-positive DCIS is associated with high-grade, larger lesion size, comedonecrosis, positive margins, and increased ipsilateral recurrence risk, and may derive greater local-control benefit from adjuvant radiotherapy. By contrast, anti-HER2 escalation in pure DCIS remains unproven, as NSABP B-43 did not meet its prespecified endpoint. Taken together, these broader data remain consistent with our central finding that biologic features, rather than BMI alone, should remain the principal framework for interpreting response and disease behavior in breast cancer [21].

Clinically, our findings indicate that BMI should not be used as a determinant of NACT candidacy or expected response. High BMI alone does not justify modification of chemotherapy strategy in stage II–III disease. Instead, emphasis should remain on tumor biology, molecular subtype, and proliferation index. Moreover, adherence to full weight-based dosing and proactive toxicity management remains essential.

### Limitations

The principal limitation is reliance on BMI rather than direct body composition assessment. BMI cannot capture visceral adiposity, sarcopenia, or metabolic dysfunction. Residual confounding related to fat distribution and metabolic phenotype cannot be excluded. Additionally, this was a retrospective single-center study; although the sample size is relatively large for a Miller–Payne-based cohort, external validation would strengthen generalizability.

## 5. Conclusions

In this large single-center cohort of non-metastatic breast cancer treated with NACT, tumor-intrinsic biological factors—particularly HER2 positivity and proliferative activity—were the dominant predictors of high Miller–Payne response. BMI was neither an independent predictor nor a significant effect modifier across receptor or menopausal subgroups. These findings suggest that anthropometric measures alone are insufficient to predict chemotherapy sensitivity and reinforce the primacy of tumor biology in guiding neoadjuvant treatment decisions.

## Figures and Tables

**Table 1:** Baseline clinicopathologic characteristics of the study cohort.

Category	Variable	Mean ± SD	Median (IQR)
Demographic	Age (years)	49.18 ± 10.94	49.00 (41.00–58.00)
Anthropometric	BMI (kg/m^2^)	28.45 ± 5.45	28.30 (24.45–31.25)
Proliferation	Ki-67 (%)	36.58 ± 25.29	30.00 (15.00–60.00)
Category	Variable	*n*	%
Clinic	Menopausal status (Postmenopausal)	307	47.45
Clinical Stage	Stage II	324	50.08
	Stage III	323	49.92
Histopathology	Invasive ductal carcinoma	578	89.34
	Other histology	69	10.66
Tumor Grade	Grade 1	59	9.12
	Grade 2	251	38.79
	Grade 3	152	23.50
Receptor Status	ER positive	340	52.55
	PR positive	176	27.20
	HER2 positive	170	26.27
Pathologic Response	Miller 1–3	382	59.04
	Miller 4–5	265	40.96

BMI, body mass index; ER, estrogen receptor; PR, progesterone receptor; HER2, human epidermal growth factor receptor 2; IDC, invasive ductal carcinoma; IQR, interquartile range; SD, standard deviation.

**Table 2:** Comparison of clinicopathologic variables according to Miller–Payne response.

Category	Miller 4–5 (*n* = 265)	Miller 1–3 (*n* = 382)	*p*-Value
Age (Mean ± SD)	48.53 ± 11.26	49.63 ± 10.70	0.299
BMI (Mean ± SD)	28.62 ± 5.94	28.33 ± 5.09	0.874
Menopausal (Post)	118 (44.53%)	189 (49.48%)	0.289
Ki-67 (%) Median (IQR)	35.00 (20.00–62.75)	25.00 (15.00–45.75)	<0.001
Clinical Stage II	146 (55.09%)	178 (46.60%)	0.034
Clinical Stage III	119 (44.91%)	204 (53.40%)	0.034
Invasive Ductal Carcinoma	238 (89.81%)	340 (89.01%)	0.008
Other Histology	27 (10.19%)	42 (10.99%)	0.008
Grade 1	11 (4.15%)	48 (12.57%)	<0.001
Grade 2	92 (34.72%)	159 (41.62%)	<0.001
Grade 3	96 (36.23%)	56 (14.66%)	<0.001
ER Positive	100 (37.74%)	240 (62.83%)	<0.001
PR Positive	40 (15.09%)	136 (35.60%)	<0.001
HER2 Positive	117 (44.15%)	53 (13.87%)	<0.001
Molecular subtype			<0.001
Luminal A-like	29 (12.2%)	89 (29.6%)	
Luminal B-like	37 (15.5%)	113 (37.5%)	
HER2-positive	117 (49.2%)	53 (17.6%)	
Triple-negative	55 (23.1%)	46 (15.3%)	

BMI, body mass index; ER, estrogen receptor; PR, progesterone receptor; HER2, human epidermal growth factor receptor 2; IQR, interquartile range.

**Table 3:** Univariable logistic regression analysis for high Miller–Payne response (Grades 4–5).

Variable	Reference	OR	95% CI	*p*-Value
Age (per year increase)	—	0.99	0.98–1.01	0.208
BMI (per kg/m^2^ increase)	—	1.01	0.98–1.04	0.508
Postmenopausal status	Premenopausal	0.84	0.60–1.16	0.289
Clinical Stage III	Stage II	0.71	0.52–0.97	0.034
Invasive ductal carcinoma	Other histology	1.09	0.66–1.79	0.740
Grade 2	Grade 1	2.52	1.25–5.10	0.010
Grade 3	Grade 1	7.48	3.59–15.58	<0.001
ER Positive (score 1)	ER 0	0.60	0.35–1.03	0.064
ER Positive (score 2)	ER 0	0.35	0.24–0.50	<0.001
ER Positive (score 3)	ER 0	1.34	0.42–4.23	0.620
PR Positive (score 1)	PR 0	0.42	0.27–0.65	<0.001
PR Positive (score 2)	PR 0	0.26	0.17–0.40	<0.001
PR Positive (score 3)	PR 0	0.72	0.32–1.58	0.410
HER2 Positive	HER2 Negative	5.20	3.54–7.64	<0.001
Ki-67 (per 1% increase)	—	1.02	1.01–1.03	<0.001

OR, odds ratio; CI, confidence interval; BMI, body mass index; ER, estrogen receptor; PR, progesterone receptor; HER2, human epidermal growth factor receptor 2.

**Table 4:** Multivariable logistic regression analysis for high Miller–Payne response.

Variable	Reference Category	Adjusted OR	95% CI	*p*-Value
Age (per year increase)	—	0.97	0.93–1.00	0.057
BMI (per kg/m^2^ increase)	—	1.00	0.96–1.05	0.964
Postmenopausal status	Premenopausal	0.37	0.17–0.81	0.013
Clinical Stage III	Stage II	0.88	0.56–1.37	0.563
Invasive ductal carcinoma	Other histology	2.86	0.94–8.33	0.063
ER Positive (score 1)	ER 0	0.82	0.34–1.96	0.656
ER Positive (score 2)	ER 0	0.50	0.25–0.98	0.043
ER Positive (score 3)	ER 0	2.10	0.29–15.04	0.459
PR Positive (score 1)	PR 0	0.89	0.46–1.76	0.747
PR Positive (score 2)	PR 0	0.57	0.29–1.13	0.109
PR Positive (score 3)	PR 0	1.02	0.31–3.41	0.971
HER2 Positive	HER2 Negative	3.71	2.23–6.20	<0.001
Ki-67 (per 1% increase)	—	1.01	1.00–1.02	0.009

aOR, adjusted odds ratio; CI, confidence interval; BMI, body mass index; ER, estrogen receptor; PR, progesterone receptor; HER2, human epidermal growth factor receptor 2; AUC, area under the curve.

**Table 5:** Adjusted interaction analysis between BMI and clinicopathologic variables for high Miller–Payne response.

Interaction Term	Events/Non-Events	Interaction OR	95% CI	Wald *p*	LR χ^2^ (df = 1)	LR *p*
BMI × Menopausal Status	265/382	0.73	0.50–1.06	0.097	2.80	0.094
BMI × HER2 Status	265/382	1.17	0.80–1.71	0.410	0.68	0.408
BMI × ER Positivity	265/382	0.80	0.56–1.13	0.207	1.61	0.205
BMI × PR Positivity	265/382	0.78	0.50–1.23	0.284	1.16	0.282

OR, odds ratio; CI, confidence interval; LR, likelihood ratio; BMI, body mass index; HER2, human epidermal growth factor receptor 2; ER, estrogen receptor; PR, progesterone receptor.

## Data Availability

The raw data supporting the conclusions of this article will be made available by the authors on request.

## Supplementary Materials

The following supporting information can be downloaded at: https://www.mdpi.com/article/10.3390/jcm15093423/s1, Table S1. Stratified Effect of BMI (per +5 kg/m^2^) on High Miller–Payne Response.
