# Supplementary material for: Body Mass Index Does Not Predict High Miller–Payne Response to Neoadjuvant Chemotherapy in Stage II–III Breast Cancer: A Single-Center Retrospective Cohort Study of 647 Patients
Source: J Clin Med. 2026 Apr 30;15(9):3423. doi: 10.3390/jcm15093423 (PMC13163852; doi:10.3390/jcm15093423)
Supplement: Supplementary file 1 [file jcm-15-03423-s001.zip › jcm-4233141-supplementary.pdf]

Supplementary Table S1. Stratified Effect of BMI (per +5 kg/m²) on High Miller–Payne Response

**Interaction Model**	**Subgroup**	**OR**	**95% CI**	***p*-value**
BMI × Menopause	Premenopausal	1.21	0.93–1.57	0.154
BMI × Menopause	Postmenopausal	0.88	0.66–1.16	0.364
BMI × HER2	HER2 Negative	0.98	0.78–1.25	0.893
BMI × HER2	HER2 Positive	1.15	0.85–1.57	0.362
BMI × ER	ER Negative	1.15	0.89–1.49	0.295
BMI × ER	ER Positive	0.92	0.71–1.19	0.504
BMI × PR	PR Negative	1.07	0.87–1.32	0.510
BMI × PR	PR Positive	0.84	0.56–1.26	0.398

OR, odds ratio; CI, confidence interval; BMI, body mass index; HER2, human epidermal growth factor receptor 2; ER, estrogen receptor; PR, progesterone receptor.
